# Supplementary material for: Fitness landscapes of human microsatellites
Source: PLoS Genet. 2024 Dec 30;20(12):e1011524. doi: 10.1371/journal.pgen.1011524 (PMC11734926; doi:10.1371/journal.pgen.1011524)
Supplement: S1 Table — (DOCX) [file pgen.1011524.s001.docx]

| **Allele size** | **Dinucleotide mutation rate** | **Trinucleotide mutation rate** |
| --- | --- | --- |
| 1x | 1 x 10^-8^ | 1 x 10^-8^ |
| 2x | 1 x 10^-8^ | 1 x 10^-8^ |
| 3x | 1 x 10^-8^ | 1 x 10^-8^ |
| 4x | 1 x 10^-8^ | 5 x 10^-7^ |
| 5x | 1 x 10^-8^ | 1 x 10^-7^ |
| 6x | 5 x 10^-8^ | 5 x 10^-6^ |
| 7x | 1 x 10^-7^ | 1 x 10^-6^ |
