# Supplementary material for: Fitness landscapes of human microsatellites
Source: PLoS Genet. 2024 Dec 30;20(12):e1011524. doi: 10.1371/journal.pgen.1011524 (PMC11734926; doi:10.1371/journal.pgen.1011524)
Supplement: S2 Table — Table A. Dinucleotide confusion matrix. Row headings are the model simulated while column headings are the predicted model. Bold-faced numbers indicate prediction accuracy for a given model, while underlined numbers mark the most common misclassification for a given model. Although false prediction rates are high for all four selection models, the primary source of confusion is distinguishing additive from dominant selection. Detection of neutral vs. non-neutral evolution is considerably more accurate. Table B. Trinucleotide confusion matrix. Notation is the same as that in Table 1a. Bold-faced numbers indicate prediction accuracy for a given model, while underlined numbers mark the most common misclassification for a given model. Although false prediction rates are high for all four selection models, the primary source of confusion is distinguishing additive from dominant selection. Detection of neutral vs. non-neutral evolution is considerably more accurate. (DOCX) [file pgen.1011524.s002.docx]

Table A.

| **ABC-RF**  **prediction**  **truth** | *Neutral* | *Additive*  *Single* | *Dominant*  *Single* | *Additive*  *Periodic* | *Dominant*  *Periodic* | False  Prediction (%) |
| --- | --- | --- | --- | --- | --- | --- |
| *Neutral* | **0.938** | 0.034 | 0.008 | 0.018 | 0.003 | 6.2 |
| *Additive Single* | 0.003 | **0.529** | 0.093 | 0.322 | 0.053 | 47.1 |
| *Dominant Single* | 0.008 | 0.110 | **0.497** | 0.037 | 0.348 | 50.3 |
| *Additive Periodic* | 0.004 | 0.235 | 0.042 | **0.613** | 0.105 | 38.6 |
| *Dominant Periodic* | 0.016 | 0.051 | 0.227 | 0.093 | **0.612** | 38.8 |

Table B.

| **ABC-RF**  **prediction**  **truth** | *Neutral* | *Additive*  *Single* | *Dominant*  *Single* | *Additive*  *Periodic* | *Dominant*  *Periodic* | False  Prediction (%) |
| --- | --- | --- | --- | --- | --- | --- |
| *Neutral* | **0.949** | 0.020 | 0.019 | 0.009 | 0.004 | 5.1 |
| *Additive Single* | 0.003 | **0.562** | 0.073 | 0.301 | 0.061 | 43.8 |
| *Dominant Single* | 0.008 | 0.053 | **0.533** | 0.023 | 0.383 | 46.7 |
| *Additive Periodic* | 0.006 | 0.268 | 0.042 | **0.570** | 0.115 | 43.0 |
| *Dominant Periodic* | 0.015 | 0.030 | 0.240 | 0.069 | **0.647** | 35.3 |
