# Supplementary figures and images for: Fitness landscapes of human microsatellites
Source: PLoS Genet. 2024 Dec 30;20(12):e1011524. doi: 10.1371/journal.pgen.1011524 (PMC11734926; doi:10.1371/journal.pgen.1011524)

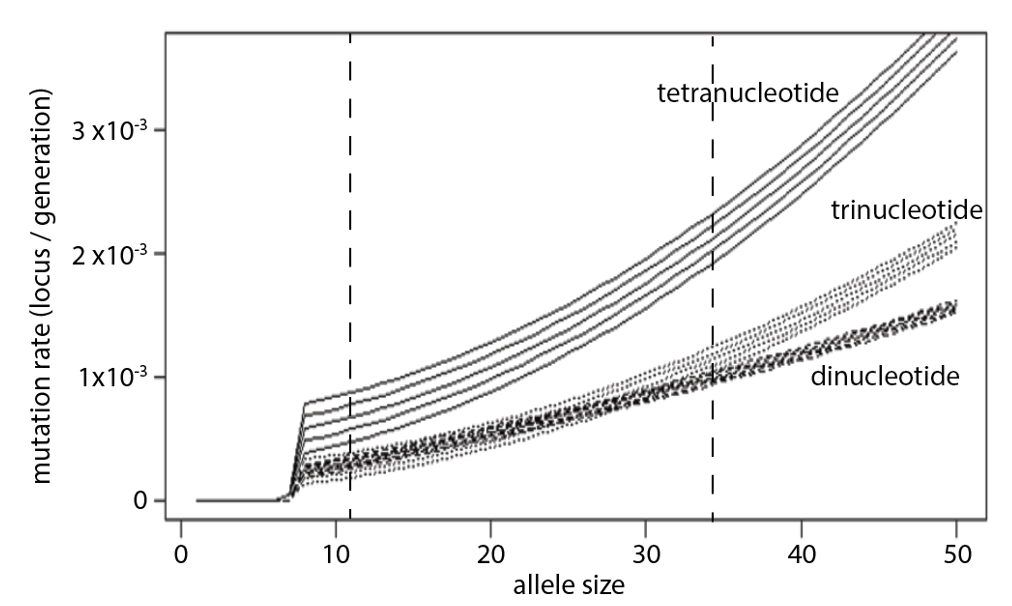

Supplement: S1 Fig — Alleles of greater size are more mutable. Each microsatellite motif size is represented by five curves to capture uncertainty and variability in mutation rate. The most reliable curves are those for dinucleotides and tetranucleotides, where the middle curve of each set was empirically estimated by Sun et al. [11] based only on alleles with sizes within the shown bounds (vertical, dashed lines). These curves begin at allele size 8. For allele sizes <7, we assumed very low mutation (μ<10−6) for all motif sizes. The tetranucleotide mutation curves are based on estimates of Sun et al. [11] and are included for comparison. (TIF) [file pgen.1011524.s003.tif]

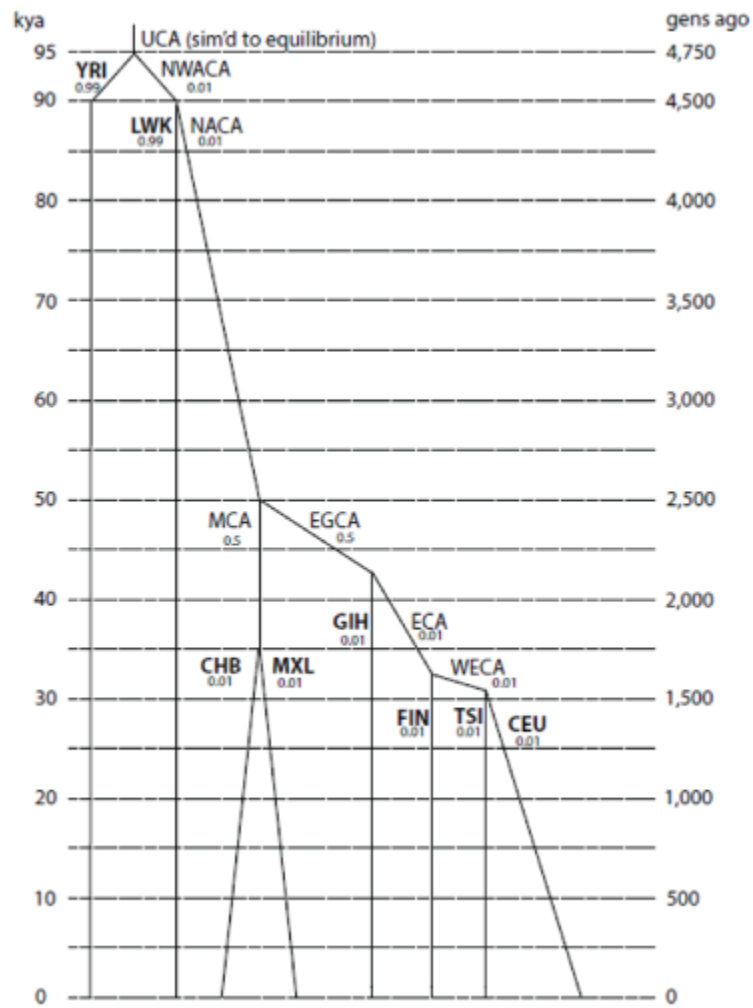

Supplement: S2 Fig — NWACA = Non-West-African common ancestor; NACA = non-African common ancestor; MCA = MXL/CHB common ancestor, EGCA = European/GIH common ancestor; ECA = European common ancestor; WECA = West-European common ancestor. The eight extant human populations sampled are in bold face. (PDF) [file pgen.1011524.s004.pdf]

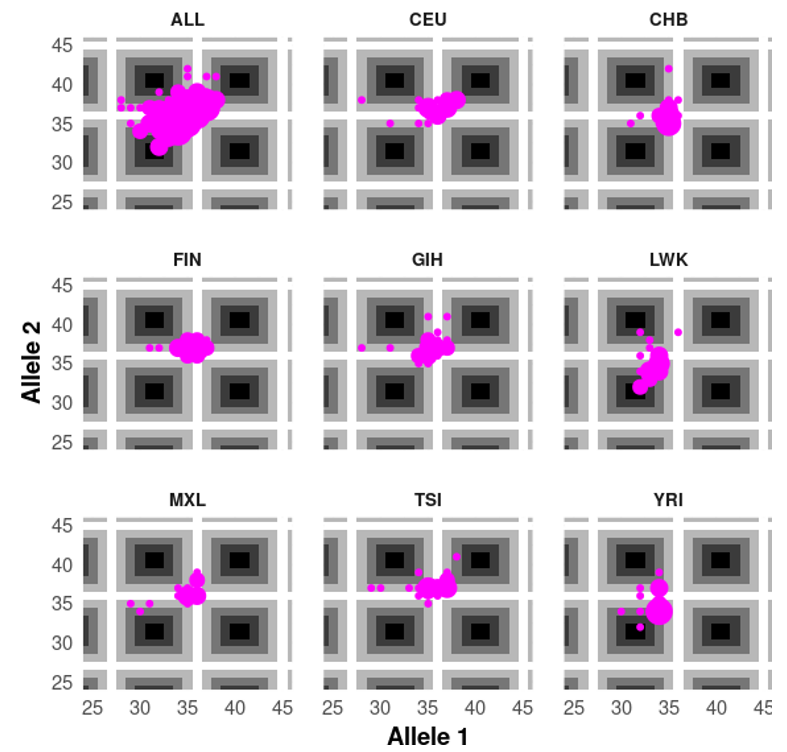

Supplement: S3 Fig — The fitness surface is overlain with the observed genotype counts (magenta circles) for all eight sampled populations and the full sample (ALL). Each population shows unexpectedly low variation in allele size given the overall average allele size of ~36. The two African populations (LWK, YRI) show a distribution of distinctly lower allele sizes. Note the bright white “ridges” on the fitness surface, which correspond to all genotypes with at least one allele whose size is divisible by 9 and that therefore have a relative fitness of 1.0. (TIF) [file pgen.1011524.s005.tif]

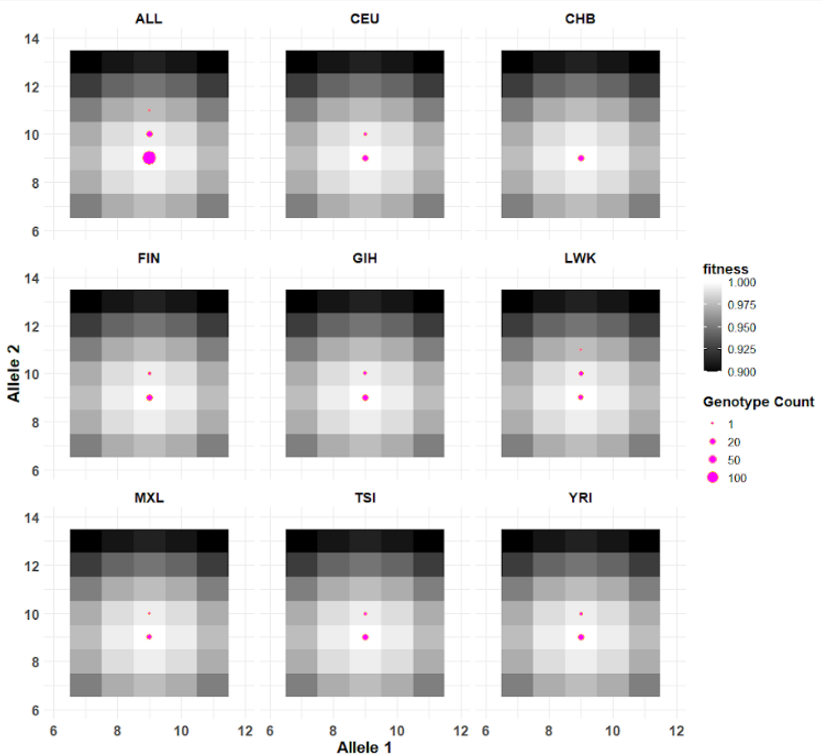

Supplement: S4 Fig — The fitness surface is focused on the only peak where observed genotypes were found in the sample. Note that heterozygous genotype counts in this figure and the following three figures are only drawn to the left of the surfaces’ antidiagonals. (TIF) [file pgen.1011524.s006.tif]

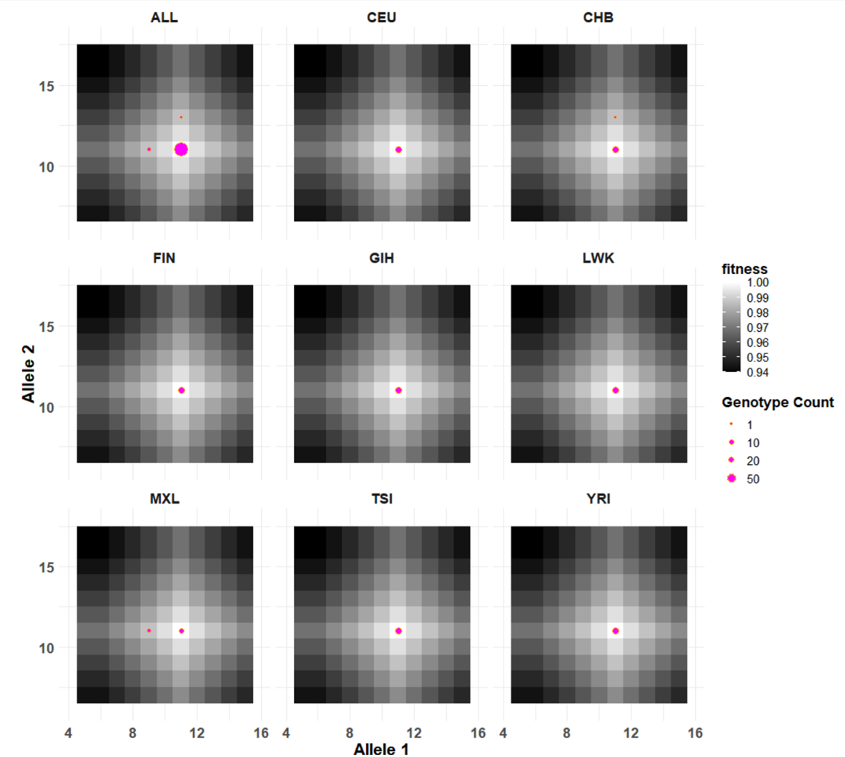

Supplement: S5 Fig — The fitness surface is focused on the only peak where observed genotypes were found in the sample. (TIF) [file pgen.1011524.s007.tif]

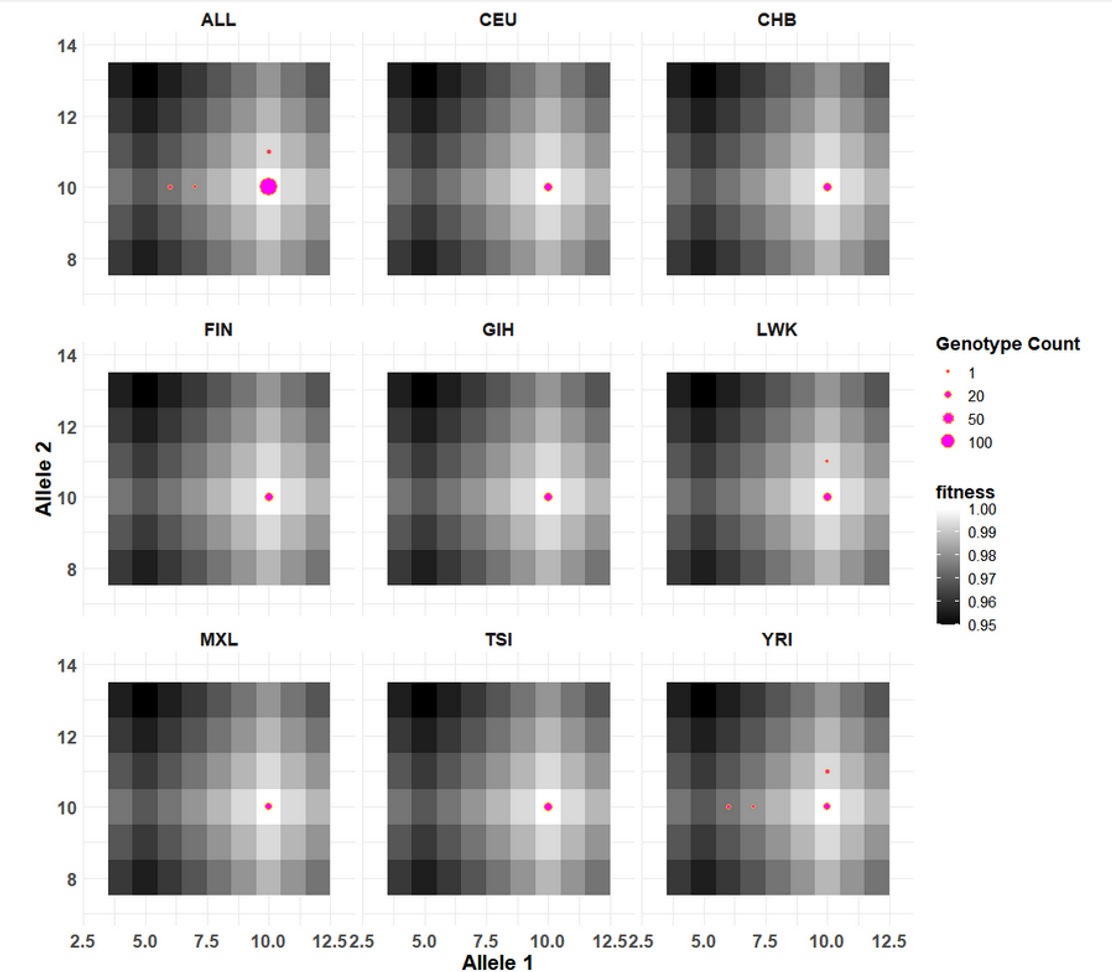

Supplement: S6 Fig — Despite the reasonably large modal allele size of 10x, variation is limited to three individuals from the African populations of LWK and YRI out of the 200 individuals sampled worldwide. This extreme invariability led to an estimated value of selection parameter s = 0.013, which is the highest point estimate of s found in this study (and shared by two other loci in which selection was implicated). (TIF) [file pgen.1011524.s008.tif]

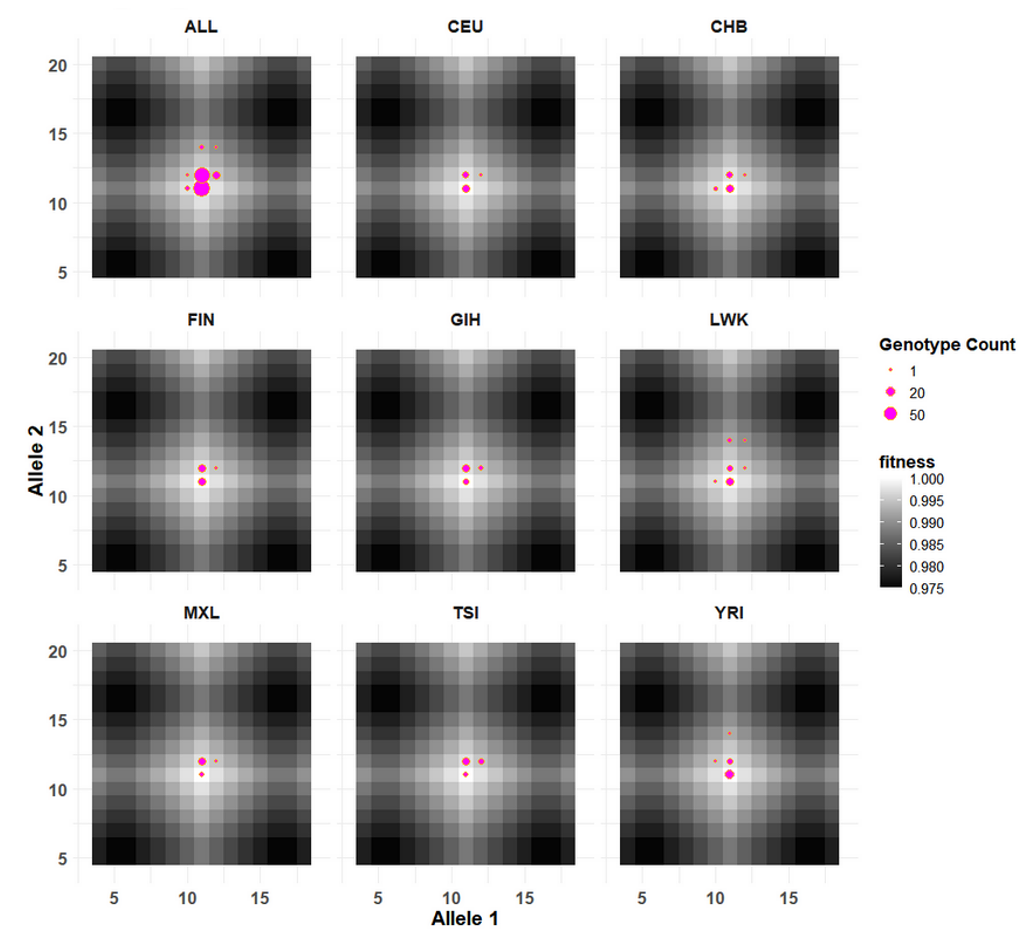

Supplement: S7 Fig — Again, we find a relatively large modal allele size (11x) but remarkably low variation (VAS = 0.298). Still, this is greater variation than that observed at the GRIN2B and DPT microsatellites. Therefore, the hill of the fitness surface slopes rather gently away from the peak at genotype 11/11 (see fitness gradient scale on right). (TIF) [file pgen.1011524.s009.tif]

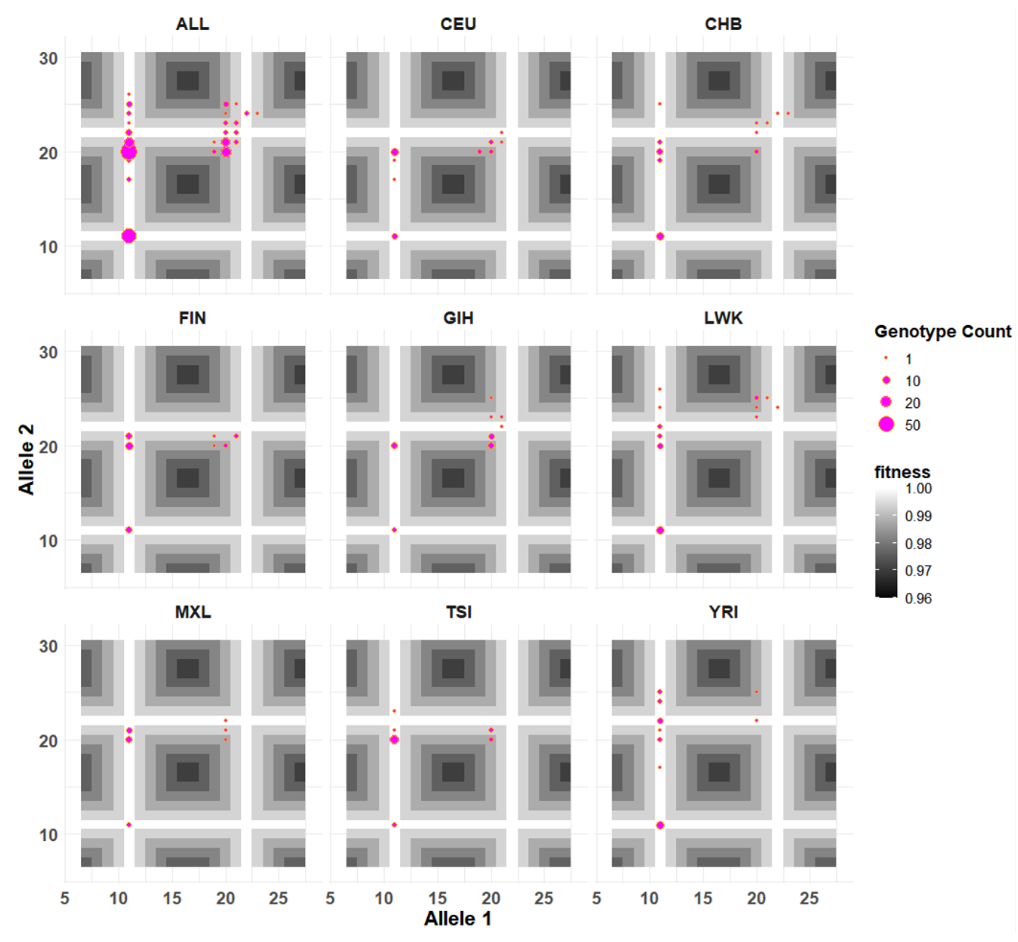

Supplement: S8 Fig — Note the evidence for dominance manifested as bright, white ridges along the fitness surface. Heterozygous genotype counts are only drawn to the left of the surfaces’ antidiagonals. (TIF) [file pgen.1011524.s010.tif]
