# Supplementary material for: Fitness landscapes of human microsatellites
Source: PLoS Genet. 2024 Dec 30;20(12):e1011524. doi: 10.1371/journal.pgen.1011524 (PMC11734926; doi:10.1371/journal.pgen.1011524)
Supplement: S1 Text — (DOCX) [file pgen.1011524.s011.docx]

**Supporting Text 1**

We obtained raw genotypes as DNA fragment sizes in base pairs. To convert fragment sizes to allele sizes, we extracted genotypes for 1000 Genomes individuals we sampled from variant call files available at <ftp://ftp.1000genomes.ebi.ac.uk/vol1/ftp/release/20130502/supporting/strs/full> based on Willems et al. (2014).

Genotypes in variant call files were provided as pairs of integers (**allele codes**) that mapped to the true **allele size**. An example is given in Table ST1.1.

**Table ST1.1.** Mapping of allele codes to true allele size at an intronic CA microsatellite (chr8:6875065-6875109, hg38 assembly) in the gene *DEFB1*. The human reference allele size of 22x, whose allele code is 0. Note that some allele codes map to “half sizes,” where 17.5x might mean 17 CAs followed by **C**G.

| **Allele code** | **Allele size** (times CA repeated) |
| --- | --- |
| 0 | 22x |
| 1 | 6x |
| 2 | 10x |
| 3 | 12x |
| 4 | 13x |
| 5 | 14x |
| 6 | 14.5x |
| 7 | 15x |
| 8 | 16x |
| 9 | 16.5x |
| 10 | 17x |
| 11 | 17.5x |
| 12 | 18x |
| 13 | 18.5x |
| 14 | 19x |
| 15 | 19.5x |
| 16 | 20x |
| 17 | 20.5x |
| 18 | 21x |
| 19 | 21.5x |
| 20 | 22.5x |
| 21 | 23x |
| 22 | 24x |
| 23 | 25x |
| 24 | 26x |

The 1000 Genomes genotype calls included numerous examples of heterozygote dropout, which means an actual heterozygote is called as a homozygote for just one of the two alleles (Table ST1.2). Careful comparison of the 1000 Genomes genotype calls to the fragment size (**raw alleles**) we obtained through electrophoresis allowed us to equate allele codes with a specific raw allele. Using this key, we converted raw alleles to true allele sizes. Table ST1.2 provides an example of this process.

**Table ST1.2.** Comparing 1000 Genomes microsatellite genotype calls (1000G_genotype) to those obtained directly from electrophoresis (raw allele 1 / raw allele 2). The *notes* column provides the logic used to infer true allele size from the fragment (raw) allele sizes. Only a few instructive examples (individuals) are shown.

| **1000G_ID** | **1000G_genotype**  (by allele code; see Table ST1.1) | **raw allele 1** (fragment size in bp) | **raw allele 2** (fragment size in bp) | **notes** |
| --- | --- | --- | --- | --- |
| HG00176 | 14/14 | 243 | 249 | The two genotypes called here are particularly misleading. Although 1000 Genomes data suggest two individuals homozygous for the same *one* allele, *four* different raw alleles were uncovered by our genotyping. This raises the obvious question: Which of the four raw alleles (if any) should we equate with allele code 14? |
| HG00181 | 14/14 | 245 | 247 |  |
| NA20846 | 12/14 | 243 | 245 | A true heterozygote suggesting **243=18x** and **245=19x**. |
| HG00380 | 16/18 | 247 | 249 | Both genotypes have an allele code of 18 and a raw allele size of 249, suggesting 18=249. We also know (Table ST1.1) that code 18 is associated with a physical allele size of 21x. This suggests we can translate fragments of size **249=21x**. |
| NA06984 | 8/18 | 243 | 249 |  |
| NA07347 | 18/18 | 249 | 251 | Heterozygote dropout in 1000 Genomes data. We’ve already inferred that 249=21x. This leaves the question: To which allele size does 251 equate? |
| NA12874 | 0/0 | 251 | 251 | A true homozygote, suggesting that allele code 0 maps to raw allele size 251, which we can extrapolate to conclude that **251=22x**. |
| NA18499 | 4/4 | 233 | 251 | Heterozygote dropout. But, if 251=22x, then the unaccounted-for raw allele of 233 must be associated with allele code 4, which means **233=13x**. |
| NA18630 | 0/0 | 245 | 251 | More examples of heterozygote dropout. Because both include a 251 raw allele, this is further evidence that 251=22x. |
| NA18631 | 0/0 | 247 | 251 |  |
| NA20505 | 16/16 | 247 | 251 | 251=22x is already established, and allele code 0 maps to 22x, suggesting that allele code 16 should be associated with raw allele 247. Again, using the information in Table ST1.1, we conclude that **247=20x**. |

Although the inferences detailed in Table ST1.2 consist of several translations from raw allele size to true, physical allele size, the sample did not include sufficient examples of each genotype, we used robust inferences to interpolate the missing pairs of raw -> true allele sizes (Table ST1.3).

**Table ST1.3.** The mapping between allele codes, raw allele sizes, and true allele sizes inferred from a comparison of electrophoretic and low coverage sequencing data. Raw allele sizes in boldface were inferred as reference calibration points following the logic noted in Table ST1.2. Raw allele sizes in parentheses indicate the allele was not observed in our sample. Raw allele sizes neither boldfaced or parenthetical were observed in our sample but not used to calibrate raw allele sizes.

| **Allele code** | **Raw allele size** (bp) | **True allele size**  (times motif repeated) |
| --- | --- | --- |
| 0 | **251** | 22x |
| 1 | (227) | 6x |
| 2 | (229) | 10x |
| 3 | (231) | 12x |
| 4 | **233** | 13x |
| 5 | (235) | 14x |
| 6 | (236) | 14.5x |
| 7 | 237 | 15x |
| 8 | **239** | 16x |
| 9 | (240) | 16.5x |
| 10 | 241 | 17x |
| 11 | (242) | 17.5x |
| 12 | **243** | 18x |
| 13 | (244) | 18.5x |
| 14 | **245** | 19x |
| 15 | (246) | 19.5x |
| 16 | **247** | 20x |
| 17 | (248) | 20.5x |
| 18 | **249** | 21x |
| 19 | (250) | 21.5x |
| 20 | (252) | 22.5x |
| 21 | 253 | 23x |
| 22 | (255) | 24x |
| 23 | (257) | 25x |
| 24 | (259) | 26x |
