# Supplementary material for: Fitness landscapes of human microsatellites
Source: PLoS Genet. 2024 Dec 30;20(12):e1011524. doi: 10.1371/journal.pgen.1011524 (PMC11734926; doi:10.1371/journal.pgen.1011524)
Supplement: S2 Text — (DOCX) [file pgen.1011524.s012.docx]

**Supporting Text 2**

In Discussion, we note the following: “Key allele size *α* of the promoter microsatellite in *TBP* has apparently shifted from around 34 to 36 between African and non-African populations. This finding was affirmed by ABC-RF model choice of (1) advanced models of selection in which African and non-African populations were allowed to take different values of *s* and *α* over (2) the models of worldwide selection covered here.”

Figure ST2.1 shows the implied shift in key allele size from 34 to 36 in non-African populations. As noted in Discussion, this was actually a rare observation; most microsatellites we sampled showed remarkably similar genotype and allele frequency distributions across all eight sampled populations.


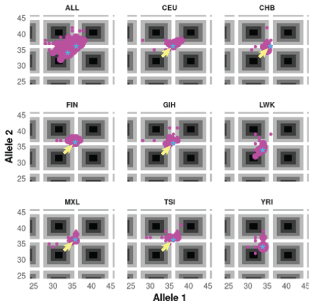


**Figure ST2.1.** Figure S3 replicated with the following amendments: (1) Blue asterisks indicate the inferred local optimal genotype of 34/34 for the two African populations and 36/36 for the six non-African populations, and; (2) the yellow arrows indicate the suggested evolution of a new optimum for non-African populations.

The “advanced models” referred to in the preceding quotation differ from the models described in the main text of the paper in that they allow different estimates of selective strength *s* and key allele size *α* for African (LWK, YRI) and non-African populations (CEU, CHB, FIN, GIH, MXL, TSI). We ran simulations under these advanced models (for trinucleotide loci, like the *TBP* microsatellite) and appended the summary statistics from each of 500,000 advanced model simulations to the “basic” trinucleotide reference table. The reference table’s rows were shuffled, and ABC-RF model choice was performed with *nine* choices of model: neutral, additive single-optimum, additive multiple-optima, dominant single-optimum, dominant multiple-optima, additive single-optimum (advanced), additive multiple-optima (advanced), dominant single-optimum (advanced), and dominant multiple-optima (advanced).

Cross-validation used to assess the performance of the ABC-RF classifier for this larger set of nine models revealed that the true model was the most common prediction for all nine models (Table ST2.1). False prediction rates for the *eight* models of selection (Table ST2.1) were comparable to those in Table S1, where only *four* models of selection were included. Also like our main results, neutral simulations were rarely classified as one of the eight selection models (only 8.5% of the time; Table ST2.1).

**Table ST2.1.** Confusion matrix for ABC-RF classification among nine models. * = “advanced” model – i.e., allows separate values of *s* and *α* for African and non-African populations.

| **prediction →**  **truth ↓** | **neutral** | **additive single** | **dominant single** | **additive periodic** | **dominant periodic** | **additive single*** | **dominant single*** | **additive periodic*** | **dominant periodic*** | **false prediction (%)** |
| --- | --- | --- | --- | --- | --- | --- | --- | --- | --- | --- |
| **neutral** | **0.915** | 0.032 | 0.024 | 0.020 | 0.002 | 0.000 | 0.002 | 0.003 | 0.002 | 8.5 |
| **additive**  **single** | 0.001 | **0.533** | 0.082 | 0.316 | 0.042 | 0.017 | 0.003 | 0.003 | 0.002 | 46.7 |
| **dominant**  **single** | 0.003 | 0.060 | **0.550** | 0.029 | 0.345 | 0.006 | 0.003 | 0.001 | 0.003 | 45 |
| **additive**  **periodic** | 0.002 | 0.249 | 0.036 | **0.555** | 0.096 | 0.008 | 0.002 | 0.015 | 0.036 | 44.5 |
| **dominant periodic** | 0.006 | 0.026 | 0.261 | 0.052 | **0.584** | 0.002 | 0.006 | 0.008 | 0.053 | 41.6 |
| **additive**  **single*** | 0.001 | 0.021 | 0.008 | 0.012 | 0.001 | **0.541** | 0.143 | 0.208 | 0.065 | 45.9 |
| **dominant**  **single*** | 0.003 | 0.005 | 0.018 | 0.006 | 0.005 | 0.055 | **0.623** | 0.010 | 0.276 | 37.7 |
| additive periodic* | 0.003 | 0.027 | 0.010 | 0.044 | 0.011 | 0.190 | 0.040 | **0.484** | 0.192 | 51.6 |
| dominant periodic* | 0.006 | 0.005 | 0.026 | 0.013 | 0.049 | 0.019 | 0.181 | 0.039 | **0.661** | 33.9 |

When we applied the ABC-RF classifier to the *TBP* data, the additive, periodic optima model received the greatest posterior support (~0.30), while the *dominant*, periodic optima model received the second greatest support (~0.25). Note that the classification of *TBP* when including only simpler models was the dominant, periodic optima model.

We then trained ABC-RF regressors to estimate selective strength *s* and key allele size *α* for African and non-African populations. Estimates of *s* were very similar for both African and non-African populations: 0.0026 (0.001, 0.012) in the two African populations and 0.0022 (0.001, 0.01) in the six non-African populations. However, estimates of alpha showed an important difference, with alpha in YRI and LWK estimated as 8 versus 7 for the six non-African populations (both received >95% posterior support). *This provides evidence in support of a tuning shift.* Recall that 36 seems to be the favored allele in non-African populations; thus 7x9=36 agrees with the data. Similarly, 8x4=32, which is not quite the average allele size of ~34 in the pooled sample of individuals from YRI and LWK in our dataset.

Finally, we note that differences between the variables most important to estimating alpha in African and non-African populations show that the ABC-RF predictor is identifying variables that seem intuitively appropriate to estimating alpha in these two different contexts. The eight most important variables (summary statistics) to estimating alpha in African populations were the frequencies of 13x, 14x, and 29x alleles in the YRI population and the frequencies of 12x, 13x, 14x, 26x, and 29x alleles in the LWK population. Contrast this with the eight most important summary statistics for estimating alpha in the six European populations: the observed heterozygosities of all six populations as well as the frequencies of the 6x allele in TSI and the 17x allele in FIN.
