# Supplementary material for: Fitness landscapes of human microsatellites
Source: PLoS Genet. 2024 Dec 30;20(12):e1011524. doi: 10.1371/journal.pgen.1011524 (PMC11734926; doi:10.1371/journal.pgen.1011524)
Supplement: Attachment — Submitted filename: HP24_Response_PlosGenResubmit_FINAL.docx [file pgen.1011524.s013.docx]

November 13**,** 2024

Dear Associate Editor Dr. Kelly and Section Editor Dr. Fay,

Thank you for handling our manuscript entitled “Fitness landscapes of human microsatellites” for *PLoS Genetics*. We revised our manuscript after careful consideration of the feedback from Dr. Kelly and the three anonymous reviewers. Major revisions include the following.

1. We moved the Materials and Methods section before the Results section. *PLoS Genetics* submission guidelines allow authors to choose this option if justification is provided. We were motivated to make this change (1) in response to requests from the associate editor and the reviewers that our models and methods be introduced before our findings are reported, and (2) to better recognize the novelty of our models and methods. We believe this reorganization significantly enhanced the clarity and flow of the manuscript.
2. We explained our methodology in greater detail.
3. We shortened and streamlined the Discussion. We deleted multiple short sections, removed detailed findings already stated in the Results, and refocused attention on interpretations with broader significance.

We believe these revisions and others substantially improved the manuscript. We hope you agree. Attached, please find detailed responses to all comments from the associate editor and the reviewers. Thank you again for your consideration.

Sincerely,

Ryan J. Haasl

Bret A. Payseur

***Please note that line numbers mentioned throughout reference the “clean” manuscript rather than the Track Changes version that is also provided. At the request of the publisher, we added an Author Summary and legends for supporting information that were not present in the Track Changes version.***

**Comments from Associate Editor Kelly**

The paper “Fitness landscapes of human microsatellites” by Haasl and Payseur has been reviewed by three expert referees. All found merit and the study and recommended either major or minor revision. I concur with the referees in their evaluation. If the authors can address their suggestions, which go mainly to increasing the clarity of the presentation, a suitably revised paper would make a fine contribution to PLoS Genetics.

In addition to the comments of the referees, I would emphasize two points for the authors to consider in revisions:

I. I agree with the suggestion from reviewer 2 that the reader needs more information about the selection models before the Results start with choosing the best model for each locus. Some combination of figures 5-6 would make a good figure 1 to sit between the Introduction and Results (preceding Table 1).

**We agree. Our failure to introduce the selection models prior to the Results section, as well as some other concerns about flow and clarity raised by reviewers, stemmed from presentation of the Results before the Materials and Methods section. To address these issues, we moved the Materials and Methods ahead of the Results. *PLoS Genetics* submission guidelines state that authors may choose this option as long as they justify it. Our primary justification for this decision is that our manuscript describes a new method for characterizing selection on microsatellites. Consequently, the novelty of the approach warrants a higher placement of the Materials and Methods section in the paper. With this change, the selection models are described before the best-fitting models (and other findings) are presented. In addition, we think the reordering of sections substantially improves clarity and flow. Please note that the reordering of sections is not marked in the Track Changes version of the revised manuscript.**

**[Relevant guidelines section from the journal: *Uniformity in format facilitates the experience of readers and users of the journal. To provide flexibility, however, authors are also able to include the Materials and Methods section before the Results section or before the Discussion section.*]**

II. Until reading this paper, I was not familiar with the ABC-RF method – I may not be alone in this regard. In most areas of biology, Bayesian methods are used more frequently for parameter estimation than hypothesis testing, the latter being difficult because Bayes factors are strongly dependent on priors. Is this not true with ABC-RF classification? If not, why not?

**We understand the concern. Early implementations of approximate Bayesian computation (ABC) model choice were criticized on these and other grounds by Robert et al. (2011, *PNAS*). In fact, these authors found that insufficiency of summary statistics can lead to significant information loss, resulting in unreliable approximations of Bayes factors and, consequently, unreliable model choice. Robert et al. (2011) therefore demonstrated that in addition to concerns regarding the influence of prior distribution choice, the very way in which ABC approximates posterior distributions can result in poor model choice outcomes.**

**The good news is that the same research group subsequently developed the ABC-RF approach to ABC model choice (Pudlo et al. 2015, *Bioinformatics*), which sidesteps both issues: prior sensitivity and loss of information due to insufficient summary statistics. In ABC-RF, rather than comparing simulations to data using a distance metric, the random forests (RF) machine learning algorithm learns the relationship between summary statistics and each model. This learning-based approach is less influenced by the priors because the RF classifier predicts model probabilities based on patterns in the summary statistics themselves, not on prior-derived likelihoods. Additionally, RF ranks the summary statistics based on their predictive power, thus reducing the need to preselect summary statistics in hopes of achieving near-sufficiency – a common challenge in traditional ABC.**

**Like all forms of ABC, the RF-based model choice approach presented by Pudlo et al. (2015) eliminates explicit likelihood calculation. No Bayes factors are calculated, and using an uninformative prior distribution on the models further minimizes potential prior influence on model choice.**

**To address the questions raised by the associate editor, we made important edits to the subsection “ABC-Random Forests Analysis” of Materials and Methods, including: (1) a clearer description of how ABC-RF differs from other approaches to approximate Bayesian computation (paragraph starting at line 376), and (2) an explanation as to why we are not concerned about the influence of prior distributions on ABC-RF model choice (edits at lines 407-411).**

Importantly, the authors get very good classification by ABC-RF when applied to simulated datasets (lines 187-200). Were the bounds on the uniform priors (lines 726-729) tuned based on the simulations or does this not matter? It may be that the authors have covered this topic in prior papers. Regardless, a paragraph (or even half a paragraph) in the Introduction of a revised paper would help the general reader to understand the method.

**We agree that we should provide more detail on how we chose the bounds on the uniform priors. We added a paragraph (beginning at line 256) that describes the rationale for the prior bounds we chose to the “Natural selection” piece within the subsection “Evolutionary Modeling” of the Materials and Methods.**

**Comments from Reviewer #1**

The authors present a method to test for selection on microsatellites. They then apply their method to a set of 94 microsatellite loci genotyped in 200 (human) individuals from eight populations.

Major revisions:

1. There should be some evaluation of how well the method works. On lines 713-714 the authors write that the method was applied to loci suspected of selection as well as putatively neutral intergenic loci. Of the 94 loci tested, 40 were suspected of selection and 54 were the putatively neutral intergenic loci. The method found that 27 of the 40 loci suspected of selection were under selection, and the method also found that 18 of the 54 putatively neutral loci were under selection. I appreciate that these are real data, and some of the loci suspected of selection might in fact be neutral and some of the putatively neutral loci might in fact be under selection (as the authors argue on lines 621-631). However, without an evaluation of the method where we have high confidence of the correct answer it is difficult to know what fraction of the results are true positives, false positives, true negatives, or false negatives. I suggest the authors evaluate their method by applying it on simulated data. They should apply their method both on neutrally simulated data and on data simulated under varying strengths of selection.

**We agree that the performance of new methods should be evaluated in known scenarios before the methods are applied to real data. As the reviewer suggests, testing our method on simulated data is an appropriate approach. We detail our measurements of method performance on simulated data in the subsection of the Results entitled “Method Performance” (beginning on line 488 of the revised manuscript). In addition to the textual summaries of performance in this subsection, Table S2 includes confusion matrices for dinucleotide and trinucleotide loci. These confusion matrices show the miscall rates for all possible types of miscalls (e.g., how often a neutrally simulated locus is misidentified as following the dominant, periodic optima model).**

**Although we believe these results sufficiently capture method performance using the approach suggested by the reviewer, we failed to mention our performance tests in the Materials and Methods section using out-of-bag error. We now do so. Please see lines 435-440 of the revised manuscript.**

2. There needs to be more clarity about imperfect repeats, e.g., (AC)7(AT)(AC)3. On lines 698-700 the authors write that microsatellite genotypes were determined by fragment-length analysis and Sanger sequencing was used to corroborate these genotypes at ten randomly chosen loci. On line 639 the authors wrote that only a few of the microsatellites they surveyed contained imperfect repeats. On lines 377-378 the authors write one of their case study microsatellites was an imperfect repeat. The authors do not say (but need to) how many individuals they used Sanger sequencing to corroborate the microsatellite genotypes. If they only used a few individuals (on only 10 microsatellites) I do not see how they can confidently say they have only a few imperfect repeats, esp. since one of their case-study microsatellites was an imperfect repeat.

**The question of imperfect repeats is an important one. We know that two of the 94 loci chosen for this study have imperfections in the reference human genome. We added details of these two imperfect repeats as footnotes to Table 1 (lines 587-589).**

**Still, we understand that the reviewer is more directly concerned with the possibility of polymorphism of perfect/imperfect repeats within human populations that we are missing. It is true that we only corroborated the genotypes of 10 loci by Sanger sequencing. Although we cannot guarantee that our repeat-length based genotypes at the other 84 loci are devoid of cryptic imperfections, we believe this is an unlikely possibility. Sequencing did not identify a single missed imperfection in the >10% of loci assessed. Furthermore, none of the fragment sizes we recovered were out of register at any of the 94 loci. For example, if we established that a 278bp fragment was a 10x allele at a dinucleotide locus, then all fragments identified were +/- 2n base pairs from 278. This observation does not exclude the possibility of “point imperfections,” but it does rule out certain indel imperfections. Overall, we have little reason to believe there are cryptic imperfections in our data set.**

**Yet, the reviewer is correct to point out that the reader deserves a transparent accounting of our treatment of imperfections, as any that exist would likely affect the mutation rate of the locus under consideration. We have added text at lines 175-177 clarifying that no imperfections were found in the loci we assessed using Sanger sequencing.**

Minor revisions:

1. There are some problems with the Figure 2 legend that need to be fixed. Part C is mentioned twice (once as a dominant single-optimum and once as a dominant periodic-optima) while part H is never mentioned. Also, the first time part C is mentioned in the figure legend it is referred to as a dominant single-optimum, while in Figure 2, part C is clearly a periodic-optima.

**We thank the reviewer for their attention to detail. We have corrected the misattributions in the figure caption, which is now Figure 5 due to the reordering of Methods and Results (lines 650-656).**

2. There needs to be more discussion of the motivation of the periodic-optima models. On lines 771-773 the authors write the periodic-optima models were motivated by the frequent occurrence of bimodal allele frequency distributions among the loci we sampled and genotyped. In several figures in the paper, I see the bimodal distributions but I don’t understand why the authors think there would be many more peaks at periodic intervals. Is this model just a way to get two optima without introducing an additional model parameter? (Perhaps mention in Discussion; address at mentioned lines if appropriate).

**This is an insightful observation. One of our motivations was, indeed, to avoid the introduction of another parameter. The periodic optima models are really a blend of empirical evidence and hypothesis/possibility. Several studies have shown the importance that spacing of cis-regulatory elements plays in the determination of gene expression level. Most notably, Vinces et al. (2009, *Science*) demonstrated that the length of repetitive elements in *S. cerevisiae* promoters directly impacts expression level, with certain allele sizes maximizing expression. Our periodic models are also an attempt to reflect the importance and selective pressure of element spacing, which microsatellites directly alter when mutated, and to allow for the possibility that there are multiple optima at regular multiples of some optimal distance.**

**We agree that the reader deserves greater detail and clarity regarding (1) the motivation for these models, (2) the limitations of what they can uncover, and (3) caution against overinterpreting the models (placing too much stock in the estimated fitness of genotypes in ranges far from the empirically observed allele sizes). Please see lines 265-269 for these changes.**

3. In the abstract on line 29, the authors write that 43 microsatellites were found to be under selection. On line 231, they write that 45 microsatellites were found to be under selection.

**Thank you. The correct number is 43. We have changed the 45 to 43 and the accompanying percentage on line 535.**

**Comments from Reviewer #2**

In this manuscript Haasl and Payseur assess 146 “carefully chosen” microsatellite loci for their potential fitness effects. They characterize the detailed fitness landscapes of these loci assuming evolution under four different selection models compared to those that evolve neutrally. The manuscript is extremely interesting and tackles a challenging problem. While it starts off extremely well written and clear in the introduction, the main text is somewhat hard to follow. Overall while this paper should almost certainly be published, I think the authors need to reorganize and rework the text to make it more streamlined and clearer. I am suggesting a major revision so the authors can take the time to make the manuscript more readable.

**We appreciate the reviewer’s concerns regarding readability. In response, we made the following changes:**

1. **We switched the order of presentation so that the Materials and Methods section is placed before the Results section. We believe this change substantially improves clarity and flow. With this shift, our models and statistical methods are described before our findings are reported, ameliorating a key issue with the first version of the manuscript.**
2. **We followed the specific suggestions provided by the reviewer below.**

Some suggestions follow:

The order of text right off the bat is a bit odd. There is some quick discussion about the distribution of the genotypes before talking about how loci were even selected. This seems out of order.

**We agree with the reviewer. In fact, this is a good example of how moving the Materials and Methods section before the Results section aids clarity. Now, with the Materials and Methods section first, the reasoning behind our choice of loci is explained in the second paragraph following the Introduction. Again, we agree this is a more natural order of presentation that we hope will facilitate readers’ understanding.**

The main text should devote more time to discussing how the “carefully curated list” was carefully curated.

**We can understand how the claim that the loci we chose were “carefully curated” might ring false in the absence of a more thorough accounting of the loci chosen. To address this problem, we first added details to the paragraph describing the reasoning behind our choice of loci in the “Data Collection” subsection of the Results in the paragraph spanning lines 179-197. Second, we replaced instances of “carefully curated” with “carefully chosen,” which is a better description of our process. In short, we aimed to dispel the notion that we believe we picked the best possible example set of loci. However, we do believe that it is a carefully chosen and representative collection of microsatellite loci.**

More up front description of the models is needed. Suggest merging Figures 6 and 7 and putting them as figure 1. These figures aren’t even really cited until the methods. The introduction should then describe the previous models published by this group (citation 58) and the main text can then provide insight into updates.

**We agree that burying the methodology in the latter portions of the paper is particularly problematic for this paper because much of its novelty (and the reader’s ability to grasp our goals) relies on the methodology. We believe moving Materials and Methods directly after the Introduction is an ideal way to provide the reader with up-front knowledge of the models and the ABC-RF approach.**

I may have missed something, but I don’t understand Fig2A given what’s written in the text. The text says a there is an optimal ridge for all genotypes with at least one 36x allele, but I see ridges for any pair where one allele is divisible by 3… what am I missing?

**The reviewer is correct that the “ridges” are not limited to any genotype with a 36x allele. Because key allele size was estimated as 9, any genotype with one allele whose repeat is divided by 9 (including 36) has maximum relative fitness of 1 (bright white on the graph). We have modified the caption to Figure S3 and the initial description of the fitness surface “ridges” at lines 667-669 to communicate these additional details.**

The discussion is similarly uneven with several short and abrupt sections followed by several reiterations of text discussed in the main. I think the discussion needs to be rewritten.

**We revised the Discussion to address both points raised by the reviewer. We deleted the first two subsections, transferring some of the material from the second one to a different subsection. We shortened the Discussion, in part by removing multiple instances in which patterns mentioned in the Results section were restated and by focusing on the implications. We think these changes significantly improved the readability of the Discussion.**

Minor comments:
Line 190 the 66% seems to give short shrift to the results which distinguish between selection and neutral much more often. Suggest some reordering.

**This is an excellent suggestion, and we have reordered the statements in the paragraph spanning lines 490-504.**

**Comments from Reviewer #3**

This is a rare paper where I don't have many super detailed questions.
It is very well written and clear.
My questions are at a higher level about some of the technical details.

1. Is there a way to test the robustness of the RF approach to assumptions about the mutation model details? Another way to phrase the question is to ask how sensitive the RF method would be to uncertainty in the empirical estimates of the mutation models?

**To address this valid concern, we used a range of mutation rate curves centered on empirical estimates (Figure S7). Each simulation began with random selection of a curve appropriate to the motif length simulated. We have added a statement to the “Evolutionary Modeling” subsection of the Results to clarify the logic of our approach. We also added a cautionary note that our inferences about selection may be less reliable for loci whose mutation rates differ dramatically from empirical estimates of microsatellite mutation rate (lines 225-228).**

2. The authors chose a subset of loci for analysis. What prevents a genome-wide analysis of "all" loci?

**Although a genome-wide analysis of all loci could ultimately be performed with our method, we focused on a modest number of loci for two reasons. First, we intentionally tackled the complicated inference of selection targeting microsatellites at a smaller scale so that we could pay close attention to how our models, simulations, and resulting fitness landscapes connected to each other. We believe the heterogeneity inherent among microsatellites provides support for choosing this path. Second, publicly available data sets of human microsatellite genotypes derived from next-generation sequencing suffer from non-trivial rates of genotyping error. For example, when using 1000 Genomes microsatellite calls to aid conversion of raw length calls into allele sizes, we noticed many loci with large percentages of miscalled genotypes (most commonly, heterozygotes called as homozygotes). See the paragraph starting at line 185 and Supplementary Text 2 for an explanation and an example of how publicly available microsatellite genotype calls derived from short-read genome sequencing can be unreliable.**

3. For the loci under selection, where do they fit in terms of contributions to background selection?

**This is a fascinating question that, in our estimation, would warrant a separate study.**

Are any in regions not inferred to experience much linked selection?

**We thank the reviewer for raising this issue. We address the concern in the third paragraph of the “Caveats and Prospects” subsection of the Discussion. Briefly, here is why we are confident our method identifies selection directly targeting microsatellites:**

a. **Linkage disequilibrium between an adaptive SNP allele rising in frequency and the microsatellite allele on which it arose decays more rapidly because of frequent mutation of the microsatellite locus in addition to recombination between the two loci.**
b. **If the linked microsatellite is polymorphic, its variance in allele size should not be drastically reduced by selection on a linked SNP allele because the SNP allele will be found linked to a diversity of microsatellite alleles. This is important because *low* variation at a microsatellite is the most common indicator of direct selection on a microsatellite.**

4. I was confused about the definition of an "x allele", such as 36x. I may have missed it but I didn't find a succinct definition in the main text. Is this simply the number of repeats?

**We thank the reviewer for pointing out that we did not clearly define this symbolism. We have now added a note to the reader explaining the meaning of this symbolism (line 627).**
